# Ligninolytic Enzyme Production and Decolorization Capacity of Synthetic Dyes by Saprotrophic White Rot, Brown Rot, and Litter Decomposing *Basidiomycetes*

**DOI:** 10.3390/jof6040301

**Published:** 2020-11-19

**Authors:** Ivana Eichlerová, Petr Baldrian

**Affiliations:** Laboratory of Environmental Microbiology, Institute of Microbiology of the Czech Academy of Sciences v.v.i., Vídeňská 1083, 142 20 Prague 4, Czech Republic; baldrian@biomed.cas.cz

**Keywords:** *Basidiomycetes*, decolorization, ligninolytic enzymes, Orange G, Remazol Brilliant Blue R

## Abstract

An extensive screening of saprotrophic *Basidiomycetes* causing white rot (WR), brown rot (BR), or litter decomposition (LD) for the production of laccase and Mn-peroxidase (MnP) and decolorization of the synthetic dyes Orange G and Remazol Brilliant Blue R (RBBR) was performed. The study considered in total 150 strains belonging to 77 species. The aim of this work was to compare the decolorization and ligninolytic capacity among different ecophysiological and taxonomic groups of *Basidiomycetes*. WR strains decolorized both dyes most efficiently; high decolorization capacity was also found in some LD fungi. The enzyme production was recorded in all three ecophysiology groups, but to a different extent. All WR and LD fungi produced laccase, and the majority of them also produced MnP. The strains belonging to BR lacked decolorization capabilities. None of them produced MnP and the production of laccase was either very low or absent. The most efficient decolorization of both dyes and the highest laccase production was found among the members of the orders *Polyporales* and *Agaricales*. The strains with high MnP activity occurred across almost all fungal orders (*Polyporales, Agaricales, Hymenochaetales,* and *Russulales).* Synthetic dye decolorization by fungal strains was clearly related to their production of ligninolytic enzymes and both properties were determined by the interaction of their ecophysiology and taxonomy, with a more relevant role of ecophysiology. Our screening revealed 12 strains with high decolorization capacity (9 WR and 3 LD), which could be promising for further biotechnological utilization.

## 1. Introduction

Many industrial applications, especially within the textile industry, represent dangerous generators of colored liquid effluent pollutants, which cause a serious hazard to the environment because of their low biodegradability. Synthetic dyes include chemically different compounds, which are classified, based on their chemical structure, as anthraquinone, azo-, phthalocyanine, triphenylmethane, or heterocyclic dyes. Their decolorization by physical and chemical methods is expensive, time-consuming, and methodologically demanding, and often not very effective. Currently, the treatment of dye effluents exploiting different microorganisms, for example, saprotrophic *Basidiomycetes*, is considered more promising [1,2].

Saprotrophic *Basidiomycetes* are considered as the most efficient decomposers of dead plant biomass in many habitats, such as deadwood, plant litter, or soil environment [3,4,5]. Because of the specific enzymatic system, they can rapidly colonize the substrate and they are capable of decomposing plant litter and wood more rapidly and more efficiently than other fungi [6,7,8,9,10]. Especially, white rot fungi are capable of degrading different xenobiotic compounds including synthetic dyes [1,11,12,13]. Moreover, the exploitation of LD fungi, which can easily colonize soil and compete with other microorganisms, in bioremediation of soil has been shown [14], yet their ligninolytic capacity is much less known. The biodegradative ability of saprotrophic *Basidiomycetes* is closely connected with the production of extracellular enzymes [15,16], among them, lignin peroxidase (EC 1.11.1.7.), laccase (EC 1.10.3.2., benzendiol/oxygen oxidoreductase), and manganese peroxidase (EC 1.11.1.13) seem to be the most important.

Laccase is a multicopper oxidase that catalyzes one-electron oxidation of wide variety of substrates, such as aromatic amines, methoxy-substitued monophenols, *o-* and *p*-diphenols, syringaldazine, and non-phenolic compounds. Manganese peroxidase represents a heme-containing enzyme catalysing oxidation of Mn^2+^ to Mn^3+^; it can oxidize a variety of phenolic substrates. Nevertheless, not only these ligninolytic enzymes participate in lignin and organopollutant degradation, other enzymatic systems (lignin peroxidase, hemedependent peroxidases, aromatic peroxygenases dye-decolorizing peroxidases, versatile peroxidases, and so on) generating hydrogen peroxide and free radicals may also be responsible for this process [17,18].

Multiple studies dealing with dye decolorization by Basidiomycetes have been published (Table 1 and Table 2), but only a few species have been studied in detail to evaluate the role of ligninolytic enzymes in the decolorization process. 

In the present paper, the ecological role and potential biotechnological exploitation of 150 strains of *Basidiomycetes* were studied, focusing on the degradation of two structurally different model dyes and the production of some ligninolytic enzymes. We directed our attention to the azo dye Orange G and the anthracene derivative RBBR (Remazol Brilliant Blue R), which is an example of an industrially important anthraquinone dye. Azo dyes, the most common and largest group of synthetic colorants, make up about a half of all known dyestuffs causing pollution in the environment all over the world [69]. They represent the most recalcitrant compounds characterized by the presence of one or more aromatic systems together with azo groups (-N = N) [70]. Anthraquinone dyes, among them RBBR, are often used as a starting chemical substance in the production of polymeric dyes, also representing an important class of organopollutants extensively used for dyeing technologies in textile and other industrial applications. The majority of these compounds are highly toxic, carcinogenic, and very resistant to degradation.

The aim of our study was to screen saprotrophic *Basidiomycetes* belonging to three ecophysiological groups for the relation between the enzyme activity and decolorization capacity of two chemically different synthetic dyes, Orange G and RBBR. The guilds of white rot, brown rot, and litter decomposing fungi were previously found to be distinct concerning the production of laccase and hydrolytic enzymes, reflecting the differences in their nutritional mode and taxonomy [71]. Such differences are also expectable with respect to their enzymatic production and may affect decolorization. We thus tried to analyze the effects of ecophysiology and taxonomy on synthetic dyes’ decolorization ability and on ligninolytic enzyme production. The screening also aimed to identify potentially efficient synthetic dye-decolorizing fungi, usable for prospective biotechnological applications. Our screening revealed 12 strains with the best decolorization properties; among them, some strains (*Lycoperdon perlatum, Oxyporus latermarginatus, Ischnoderma benzoinum*) had not been extensively studied before. Nevertheless, they could be prospective organisms for further biotechnological exploitation. 

## 2. Materials and Methods

### 2.1. Organisms

All studied strains were obtained from the Culture Collection of Basidiomycetes (CCBAS, Institute of Microbiology of the CAS v.v.i., Prague, Czech Republic). Fungal strains were maintained on malt extract agar (malt extract 2%) and kept at 4 °C. Taxonomic inclusion of fungi in higher taxa was according to Index Fungorum classification (http://www.indexfungorum.org/).

### 2.2. Decolorization Assay

A semi-quantitative agar–plate test was used for evaluation of the synthetic dye decolorization capacity in the tested set of fungi. The test was carried out on solid N-limited Kirk medium [72] in Petri dishes (90 mm diameter) supplemented with Orange G or RBBR at a final concentration of 200 mg l^−1^. Four parallels of plates were inoculated with mycelial plugs (3 mm diameter) cut from the actively growing part of mycelia on solid N-limited Kirk medium [72] and incubated at 27 °C for 21 days.

The growth rate (colony diameter) and diameter of the decolorized zone (or zone of coloure change) were measured daily. The decolorized zone was evaluated on a five-point scale: 0−20 mm = 1; 21−30 mm = 2; 31−50 mm = 3; 51−70 mm = 4; and 71−90 mm = 5. The diameter of mycelia was also evaluated on a five-point scale: 0−20% of control = 1; 21−30% of control = 2; 31−50% of control = 3; 51−70% of control = 4; and 71−100% of control = 5. Plates without dyes were used as a growth control for each strain, while uninoculated plates with the dyes were used as an abiotic controls (physicochemical decolorization). All measurements were performed in triplicates.

### 2.3. Ligninolytic Enzyme Assays

The experiments were carried out in 100 mL Erlenmeyer flasks containing 20 mL of N-limited Kirk medium [72]. The flasks, inoculated with two agar plugs (N-limited Kirk medium, 10 mm diameter) cut from the actively growing part of a colony in a Petri dish, were incubated at 27 °C for 21 days. Enzyme activity was determined daily in filtrates from four parallel flasks obtained after mycelia removal. Laccase and manganese peroxidase (MnP) enzymatic activities were measured spectrophotometrically as the absorbance increased at 425 nm (laccase) or 590 nm (MnP) in liquid culture. The method of Bourbonnais and Paice [73], based on the oxidation of ABTS (2,2’-Azino-bis(3-ethylbenzthiazoline-6-sulfonic acid), was used for the determination of laccase activity. MnP activity was followed using MBTH (3-methyl-2-benzothiazolinone hydrazone, Sigma) and DMAB (3-dimethylaminobenzoic acid, Sigma) according the method of Ngo and Lenhoff [74], modified by Daniel et al. [75]. One unit of enzyme activity (U) represents an amount catalyzing the production of one micromole of dye (green in the case of laccase and purple in the case of MnP) per milliliter per minute.

## 3. Results 

**Figure 1:**
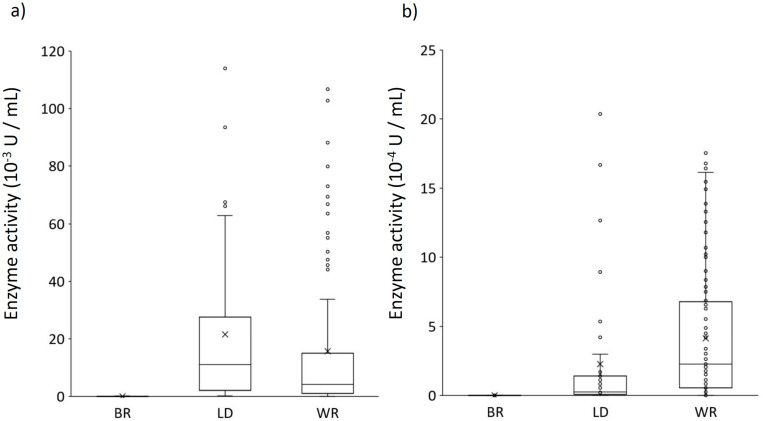
Distribution of peak activities of laccase (**a**) and Mn-peroxidase (**b**) in cultures of brown rot fungi (BR, *n* = 7), litter decomposing fungi (LD, *n* = 38), and white rot fungi (WR, *n* = 105). The box plots indicate upper and lower quartiles and means, while outlier values are represented as individual points.

One hundred and fifty strains of fungi belonging to 77 species were included in this study (Table 3) and were classified into three ecophysiological groups: litter decomposing *Basidiomycetes* (LD) and wood decomposers—white rot (WR) or brown rot (BR). The majority of the strains belonged to orders *Agaricales*, *Polyporales*, and *Hymenochaetales*. The results of the dye decolorization, fungal radial growth rate, and enzyme activities, together with ecophysiological and taxonomic characteristics of tested strains, are reported in Table 4 and Table 5 and in Figure 1a,b.

### 3.1. Agar–Plate Screening for Orange G and RBBR Decolorization

In terms of decolorization activity, 107 (71.3%) strains were active against one or both dyes within 14 days, but only 63 (58.9%) strains decolorized both Orange G and RBBR (Table 4). Among them, 34 (22.7%) strains decolorized both dyes equally, however, 15 (10.0%) strains decolorized Orange G to a higher extent (the diameter of decolorized zone was greater than 30 mm) within 14 days; in the case of RBBR, it was 14 (9.3%). The decolorization in a majority of the strains started around the fifth day of cultivation. No decolorization was observed in control plates. None of the strains decolorized only Orange G, but 44 (41.1%) strains decolorized only RBBR, while 43 (28.6%) strains did not decolorize any dye. The strains that did not show decolorization capabilities within 14 days also gave the same results after 20 days of cultivation (data not shown).

Our results revealed that WR fungi were the most efficient in dye decolorization. High Orange G decolorization capacity (the diameter of decolorized zone after 14 days was greater than 30 mm) was recorded in 38.1% of WR fungi (40 WR strains); in the case of RBBR, it was 44 strains (41.9%). High decolorization of both Orange G and RBBR was achieved by 26 strains (24.8%) of WR fungi. On the other hand, BR fungi did not decolorize either Orange G or RBBR. In the case of LD fungi, only 9 strains (23.7%) decolorized Orange G to the high extent and 11 strains (28.9%) showed high decolorization of RBBR. The majority of these fungi did not decolorize Orange G (27 strains; 71.1%), but were able to decolorize only RBBR to a high or low extent; 9 strains (23.7%) of LD fungi did not decolorize any of the dyes tested. Only 3 strains (7.9%) declorized both dyes to the high extent. Both Orange G and RBBR were most efficiently decolorized by the members of the *Polyporales* (belonging mostly to the families *Polyporaceae*, *Fomitopsidaceae*, and *Meruliaceae*) and by *Agaricales* (families *Agaricaceae*, *Pleurotaceae*, *Strophariaceae*, and *Tricholomataceae*). In *Hymenochaetales*, we also found several strains with high decolorization abilities, where the most efficient one was a member of the family *Schizoporaceae* (Table 5).

Twelve of the tested strains showed the highest decolorization of both dyes (the diameter of the decolorized zone after 14 days was > 70 mm). Nine of them represented WR: *Abortiporus biennis* (CCBAS 521), *Ischnoderma benzoinum* (CCBAS 553), *Oxyporus latermarginatus* (CCBAS 810), *Pleurotus djamor* (CCBAS 666), *Polyporus lepideus* (CCBAS 608), *Pycnoporus sanguineus* (CCBAS 595 and *Pycnoporus* CCBAS 596), *Trametes hirsuta* (CCBAS 610), and *Trametes versicolor* (CCBAS 612); and three of them were litter decomposers: *Cyclocybe erebia* (CCBAS 811), *Lycoperdon perlatum* (CCBAS 516), and *Tricholoma sejunctum* (CCBAS 750). Out of these 12 strains, four showed only high laccase activity; two of them only high MnP activity; three strains exhibited both laccase and MnP high activities; but in three strains, we found low or even no (MnP activity in one case) enzyme activity.

### 3.2. Growth on Agar Plates Containing Dyes

All studied fungi, even the strains without decolorization abilities, were able to grow on solid media in the presence of the dyes. Nevertheless, the majority of the strains grew more slowly on plates with dyes than in control plates without dyes. Only 33 strains (22%) were not sensitive to the toxicity of one or both dyes and their growth rate was comparable with the control plates.

Mostly, we have recorded a positive correlation between the radial growth rate and the decolorization. Successfully growing strains on dye-containing plates usually efficiently decolorized that dye. Radial growth rate was extremely reduced in 34 (22.6%) strains without Orange G decolorization capability and the same was found in 3 (2.0%) strains, which were not able to decolorize RBBR. Nevertheless, the radial growth rate of some strains was partly inhibited by the dye even when dye decolorization was efficient (see Table 4). This phenomenon was found only in 3 (2.0%) strains decolorizing Orange G and in 11 (7.3%) strains decolorizing RBBR. The radial growth rate in individual strains was mostly similar on both dyes, but in 42 (28%) strains, we found significantly higher growth reduction in the presence of RBBR, while 9 (6%) strains grew more slowly on medium containing Orange G.

Numerous isolates of WR (68 strains; 64.8%) and BR (5 strains; 71.4%) showed a high radial growth rate (the diameter of colony was greater than 30% of the control after 14 days of growth) on Orange G; in the case of RBBR, it was 57 strains (54.3%) of WR as well as 5 strains (71.4%) of BR. On the other hand, LD fungi seemed to be more sensitive to the dyes, especially to RBBR, which caused a significant growth reduction in 27 (71.1%) of LD strains (see Table 4).

### 3.3. Ligninolytic Enzyme Assays

All strains were also included in the enzyme activity analysis (see Table 4 and Table 5 and Figure 1a, b). In the majority of the strains, laccase and MnP achieved their highest production between the 10th and 14th day of cultivation (see Table 4). Of the fungi tested, 30 (20.0%) produced high titres of laccase (higher activity than 3 × 10^−3^ U/mL in maximum) and 51 (34.0%) strains showed high activity of MnP (higher activity than 3 × 10^−4^ U/mL in maximum); 14 (9.33%) strains exhibited high activity of both laccase and MnP. Only two (1.33%) strains did not produce laccase and 16 (10.7%) strains did not excrete MnP. We noticed the differences between the occurrence and activity of laccase in various ecophysiological and taxonomic groups. Both enzymes were recorded in WR and LD fungi, but to different extent; in BR, MnP was not recorded and laccase activities were either absent or just slightly above the detection limit (Figure 1a, b). All WR fungi showed activity of laccase, and the activity was high in 21 of them (20.0%). We found a similar situation in LD fungi, where 9 (23.7%) strains exhibited high laccase activity. The majority of WR strains (except four strains) also produced MnP; in 45 (42.9%) of them, we detected high activity of MnP. Nevertheless, only six (15.8%) LD strains showed high MnP activity, while five (13.2%) LD strains did not produce MnP at all. Out of the BR strains tested, all seven strains produced laccase. None of the BR strains showed high laccase activity and two (28.6%) BR strains did not produce laccase at all. We have often seen different and variable production of both enzymes by the members of various fungal orders. Significantly higher laccase activity was mostly found in *Agaricales* and *Polyporales*; in one case, we also detected high laccase excretion in a strain belonging to the *Russulales*. We found high MnP activity in different strains across all fungal orders, mostly in *Hymenochaetales* (57.9%) and in *Polyporales* (41.5%); however, we detected the two highest levels of MnP production in *Omphalina mutila* belonging to the *Agaricales* and in *Hericium erinaceus* from the *Russulales* (see Table 5). 

Out of 150 different basidiomycetes screened, 12 of them (Abortiporus biennis, Cyclocybe erebia, Ischnoderma benzoinum, Lycoperdon perlatum, Oxyporus latermarginatus, Pleurotus djamor, Polyporus lepideus, Pycnoporus sanguineus (two strains), Trametes hirsuta, Trametes versicolor, and Tricholoma sejunctum) showed the best decolorization properties.

## 4. Discussion

To evaluate the decolorization capacity of different saprotrophic fungi, we applied a screening method with two different dyes (azo and antraquinone dyes) differing in their chemical structure. As the azo and antraquinone dyes represent the most common dye groups, an analogous decolorization test has also been successfully used by other researchers [76,77]. Our findings confirm that the azo dye was more recalcitrant than the anthraquinonic one, which corresponds with the results reported by others authors [22,60,76,78,79,80].

In our studied set of strains, RBBR was mostly decolorized more easily than Orange G. Nevertheless, we recorded higher growth reduction in strains growing on plates with RBBR (partly even in the case of efficient RBBR decolorization), probably due to the enhancement of the toxicity of RBBR or its intermediates formed during the decolorization process [77,78]. This is consistent with our previous results [81].

We can conclude that both azo and anthraquinone dyes are toxic and resistant to biodegradation; however, individual strains of saprotrophic fungi differ from each other not only in their capacity to decolorize these dyes, but also in their sensitivity to those dyes and to the products of their degradation. Anthraquinone-based dyes, considered as a derivatives of *p*-benzoquinones, are, in consequence of their structures, very resistant to degradation [66,82]. In the case of azo dyes, for successful degradation is necessary to disrupt the azo bonds at first and further efficient degradation of aromatic rings depends on the characteristics of functional groups and on its interaction with the azo bonds [2,83,84,85].

Our results indicate that both enzymes cooperate in the decolorization process; low production of MnP may be compensated by high laccase production (e.g., *Abortiporus biennis*, *Cyclocybe erebia*), and vice versa (e.g., *Oxyporus latemarginatus*). Nevertheless, efficiently decolorizing taxa differ in laccase and MnP production, thus conclusions about the principal role of one of the enzymes cannot be drawn. Some authors reported that laccase plays the primary role in Orange G decolorization, while MnP production is more significant for RBBR decolorization [20,69,86,87,88]; nevertheless, others consider MnP to be essential for both azo and anthraquinone dye decolorization [31,89,90,91]. On the other hand, some authors declared the crucial role of laccase in the decolorization of RBBR [62,67,68] or in the decolorization of both anthraquione and azo dyes [28,49]. Although it is widely known that the ligninolytic enzymes laccase and MnP produced by saprotrophic fungi play a crucial role in the degradation of various xenobiotics, including dyes [92], the process of their degradation is more complicated and many other factors (different mediators, radicals, hydrogen peroxide, other oxidative enzymes, and so on) participate in it [7,17,18,93,94]. For example, one can mention the most recently discovered novel types of hemedependent peroxidases, aromatic peroxygenases, and dye-decolorizing peroxidases, which catalyze remarkable reactions such as peroxide-driven oxygen transfer and cleavage of anthraquinone derivatives [7,94]. Furthermore, “classic” fungal heme-containing peroxidases, i.e., lignin peroxidase and versatile peroxidases, are involved in this process. It is important to also take into account nonenzymatic degradation mechanisms, such as Fenton reactions with the aid of peroxide-producing enzymes.

To compare and characterize different ecophysiological and taxonomic groups of *Basidiomycetes*, we screened our set of fungi for laccase and MnP production under standard conditions (without dye-inducers). Significantly higher laccase activity was mostly found in *Agaricales* and *Polyporales*; however, like in our previous studies [71], our results indicated that the members of these orders were phenotypically diverse, partly because these orders include all ecophysiological groups—WR, BR, and LD fungi. We can conclude that decolorization capacity and ligninolytic enzyme production of saprotrophic fungi are determined by their ecophysiology and taxonomic position, with a more important role of ecophysiology. This is in agreement with our previous finding [71]. Our results demonstrate a positive correlation between the production of ligninolytic enzymes and synthetic dye decolorization. We recorded that, in some strains, Orange G is decolorized by laccase and RBBR by MnP; however, in other strains, we observed the opposite situation.

Much information on the decolorization of synthetic dyes by ligninolytic fungi has been obtained with *Phanerochaete chrysosporium* [11,37,39,56], *Pleurotus ostreatus* [16,49,54,57,65,68,77], *Trametes versicolor* [25,42,50,85], or *Bjerkandera adusta* [76,95,96]. Our screening showed 12 strains with the best decolorization properties. We revealed, among them, some strains (*Lycoperdon perlatum, Oxyporus latermarginatus, Ischnoderma benzoinum*) that have so far attracted only little or no research attention. Nevertheless, they are efficient dye degraders and seem to be prospective organisms for further biotechnological exploitation. Therefore, these strains will be further investigated and research efforts need to be focused on them. Research by many teams around the world shows that attempts to decolorize dyes using microorganisms in laboratory conditions were equally successful in natural, especially aquatic, environments such as decolorization of wastewater, among others. Therefore, we believe that screening of fungi in laboratory conditions is an important first step to their possible use in practice.

## 5. Conclusions

Orange G and RBBR decolorization abilities are widespread in saprotrophic fungi; among them, WR fungi is the most efficient. High decolorization capacity was also found in many LD strains, while BR fungi did not show decolorization capacity. All WR and LD fungi produced laccase and the majority of them produced MnP. Both dyes tested were the most efficiently decolorized by members of the orders *Polyporales* and *Agaricales*. These two orders also comprised the species with the highest laccase production, while high MnP production occurred in different strains across all orders studied. Synthetic dyes’ decolorization capabilities of the fungal strains are connected to their production of ligninolytic enzymes. Moreover, these properties are determined by their ecophysiology and taxonomic placement; the ecophysiology seems to be more important. Our screening revealed 12 strains with the highest decolorization capacity, some of them from genera not previously reported, which could be prospective organisms for further use in biotechnology and would deserve further investigation.

## Figures and Tables

**Table 1:** Overview of the azo-dye decolorization capacity and laccase and Mn-peroxidase (MnP) production in *Basidiomycetes.*

Organism	Dye Decolorized	Enzyme Studied	Reference
*Daedalea dickinsii*	Disperse violet-63 Disperse orange-30	Laccase, MnP	[19]
*Pleurotus sajor-caju*	Orange G	Laccase	[20]
*Oudemansiella canarii*	Congo red	Laccase	[21]
*Clitopilus scyphoides, Ganoderma resinaceum, Schizophyllum* sp.	Reactive Black 5	MnP	[22]
*Lenzites elegans*	Congo Red	Laccase	[23]
*Trametes versicolor*	Orange G, Acid Orange 6	Laccase	[24]
*Trametes versicolor*	Reactive Black 5	Laccase	[25]
*Pleurotus sajor caju*	Congo Red	MnP	[26]
*Phanerochaete chrysosporium*	Congo Red	MnP	[27]
*Marasmius cladophyllus*	Orange G	Laccase	[28,29]
	Kongo Red		
*Trametes trogii*	Reactive Black 5, Reactive Violet 5	Laccase	
*Phanerochaete sordida*	Reactive Black 5	Laccase	[30]
*Irpex lacteus*	Remazol Brilliant Violet 5R, Direct Red 5B	MnP	[31]
*Pleurotus ostreatus*	Orange II, Reactive Black 5 (RB5), (DMP), Phenol Red	MnP	[32]
*Coriolopsis gallica*	Reactive Black 5, Bismark Brown R	Laccase	[33]
*Coprinus comatus*	Reactive Dark Blue KR, Reactive orange 1, Reactive Red X-3B, Congo red	Laccase	[34]
*Hygrocybe* sp., *Lentinus bertieri*, *Lentinus villosus*, *Peniophora cinerea*, *Pleurotus flabellatus*, *Pleurotus ostreatus*, *Psilocybe castanella*, *Pycnoporus sanguineus*, *Rigidoporus microsporus*, *Trametes villosa*,	Cibacron Red	Laccase	[35]
*Pleurotus ostratus*	Orange II, Reactive Black 5, Amaranth	MnP	[36]
*Phanerochaete chrysosporium*	Direct Red 80, Mordant -9 Blue	MnP	[37]
*Pleurotus ostreatus*	Congo Red	Laccase	[38]
*Pleurotus ostreatus*	Drimarene Brilliant Red K-4BL	Laccase	[12]
*Phanerochaete. chrysosporium, Pycnoporus cinnabarinus*	Congo Red, Xylidine Ponceau 2R	Laccase, MnP	[39]
*Coriolopsis byrsina, Lentinus strigellus, Lentinus* sp., *Pycnoporus sanguineus, Phellinus rimosus*	Orange II	Laccase	[40]
*Cyathus bulleri*	Reactive Orange 1	Laccase	[41]
*Coriolopsis polyzona, Hypholoma fasciculare, Pycnoporus sanguineus, Stropharia rugosoannulata, Trametes versicolor*	Acid Red 299, Direct Blue 1, Direct Red 28, Disperse Red 1, Disperse Yellow 3, Reactive Black 5, Reactive Red 4, Reactive Yellow 81	Laccase	[42]
*Irpex lacteus*	Reactive Orange 16	MnP	[43]
*Datronia caperata, Polyporus tenuiculus, Pycnoporus sanguineus, Hexagonia hirta*	Orange II	Laccase	[44]
*Ischnoderma resinosum*	Reactive Black 5, Reactive Red 22, Reactive Yellow 15	Laccase, MnP	[45]

**Table 2:** Overview of the anthraquinone-dye decolorization capacity and laccase and MnP production in *Basidiomycetes.* RBBR, Remazol Brilliant Blue R.

Organism	Dye Decolorized	Enzyme Studied	References
*Microporus vernicipes, Peniophora incarnata, Perenniporia subacida, Phanerochaete sordida, Phlebia acerina, Phlebia radiata.*	RBBR	Laccase, MnP	[1]
*Ganoderma lucidum*	RBBR	Laccase	[46]
*Trametes hirsuta*	RBBR, Acid Blue 129	Laccase, MnP	[47]
*Ganoderma boninense, Ganoderma.miniatocinctum, Ganoderma zonatum, Ganoderma tornatum*	RBBR	Laccase, MnP	[48]
*Pleurotus ostreatus*	RBBR, Bromfenol Blue	Laccase	[49]
*Trametes versicolor*	RBBR	Laccase	[50]
*Clitopilus scyphoides, Ganoderma resinaceum, Schizophyllum* sp.	RBBR	MnP	[22]
*Ganoderma lucidum*	RBBR	Laccase, MnP	[51]
*Panus strigellus*	RBBR, Reactive Blue 220	Laccase	[52]
*Lentinus polychrous*	RBBR	Laccase	[53]
*Trametes hirsuta, Phanerochaete chrysosporium, Pleurotus ostreatus*	RBBR	Laccase, MnP	[11]
*Marasmius cladophyllus*	RBBR	Laccase	[28]
*Pleurotus ostreatus*	RBBR	Laccase	[54]
*Ganoderma* sp.	RBBR	Laccase	[55]
*Phanerochaete chrysosporium, Ceriporiopsis subvermispora*	RBBR	Laccase, MnP	[56]
*Irpex lacteus*	RBBR	MnP	[31]
*Coriolopsis gallica*	RBBR	Laccase	[33]
*Coprinus comatus*	RBBR, Reactive Brilliant Blue X-BR, Reactive Brilliant Blue K-GR, Reactive Brilliant Blue K-3R	Laccase	[34]
*Pleurotus ostreatus*	RBBR	Laccase, MnP	[57]
*Cerrena* sp.	RBBR	Laccase	[58]
*Mycena purpureofusca*	RBBR	Laccase	[59]
*Trametes hirsuta, Pycnoporus* sp., *Irpex* sp.	RBBR	Laccase	[60]
*Polyporus* sp.	RBBR	Laccase	[61]
*Ganoderma lucidum*	RBBR	Laccase	[62]
*Pycnoporus* sp.	RBBR	Laccase	[63]
*Fomes fomentarius*	RBBR	Laccase	[64]
*Pleurotus ostreatus*	Drimarene Blue K2RL	Laccase, MnP	[65]
*Lentinus crinitus, Psilocybe castanella*	RBBR	Laccase	[66]
*Coriolopsis byrsina, Lentinus strigellus, Lentinus* sp., *Pycnoporus sanguineus, Phellinus rimosus*	RBBR, Cibacron Blue 3GA	Laccase	[40]
*Coriolopsis polyzona,* *Hypholoma fasciculare, Pycnoporus sanguineus, Stropharia rugosoannulata, Trametes versicolor*	Acid Blue 62, Disperse Blue 1, Reactive Blue 19	Laccase	[42]
*Irpex lacteus*		MnP	[43]
*Ischnoderma resinosum*	Reactive Blue 19	Laccase, MnP	[45]
*Pycnoporus sanguineus*	RBBR	Laccase	[67]
*Pleurotus ostreatus*	RBBR	Laccase	[68]

**Table 3:** Overview of the ecophysiological and taxonomical classification of the studied *Basidiomycetes.*

Ecophysiological Classification	Distribution of Taxonomical Groups in the Studied Set (*n*)				
	Orders	Families	Genera	Species	Strains
White rot	4	13	33	52	105
Brown rot	2	3	4	4	7
Litter decomposers	1	7	15	21	38

**Table 4:** Decolorization capacities and ligninolytic enzyme production in saprotrophic *Basidiomycetes.* Abbreviations: WR—white rot, BR—brown rot, LD—litter decomposers.

CCBAS Number	Genus	Species	Family	Order	Ecology	Lacc ^1^	MnP ^2^	Orange G		RBBR	
								Decolorization ^3^	Growth Rate ^4^	Decolorization ^3^	Growth Rate ^4^
**521**	*Abortiporus*	*biennis*	*Meruliaceae*	*Polyporales*	WR	102.73	0.94	5	5	5	5
**498**	*Abortiporus*	*biennis*	*Meruliaceae*	*Polyporales*	WR	88.25	0.61	4	4	4	4
**301**	*Agaricus*	*arvensis*	*Agaricaceae*	*Agaricales*	LD	8.23	0.28	0	3	0	2
**643**	*Agrocybe*	*smithii*	*Strophariaceae*	*Agaricales*	LD	24.74	0.09	2	2	3	2
**803**	*Agrocybe*	*smithii*	*Strophariaceae*	*Agaricales*	LD	42.96	0.09	3	4	3	2
**641**	*Agrocybe*	*praecox*	*Strophariaceae*	*Agaricales*	LD	12.71	0.56	1	1	2	1
**543**	*Antrodia*	*heteromorpha*	*Fomitopsidaceae*	*Polyporales*	BR	0.14	0.00	0	5	0	4
**542**	*Antrodia*	*heteromorpha*	*Fomitopsidaceae*	*Polyporales*	BR	0.20	0.00	0	5	0	4
**706**	*Antrodia*	*heteromorpha*	*Fomitopsidaceae*	*Polyporales*	BR	0.04	0.00	0	4	0	3
**331**	*Armillaria*	*gemina*	*Physalacriaceae*	*Agaricales*	WR	5.50	1.50	0	1	1	1
**330**	*Armillaria*	*calvescens*	*Physalacriaceae*	*Agaricales*	WR	4.87	2.72	0	1	1	1
**833**	*Armillaria*	*calvescens*	*Physalacriaceae*	*Agaricales*	WR	6.86	0.84	0	1	1	1
**558**	*Ceriporia*	*camaresiana*	*Phanerochaetaceae*	*Agaricales*	WR	4.27	0.56	1	3	2	3
**678**	*Armillaria*	*sinapina*	*Physalacriaceae*	*Agaricales*	WR	3.11	0.09	0	1	0	1
**344**	*Clitopilus*	*passeckerianus*	*Entolomataceae*	*Agaricales*	LD	36.03	12.67	3	4	2	2
**775**	*Clitopilus*	*passeckerianus*	*Entolomataceae*	*Agaricales*	LD	66.15	8.94	4	4	2	4
**775**	*Clitopilus*	*passeckerianus*	*Entolomataceae*	*Agaricales*	LD	62.80	4.22	3	3	4	4
**356**	*Coprinellus*	*bisporus*	*Psathyrellaceae*	*Agaricales*	LD	13.24	0.84	0	2	1	1
**357**	*Coprinellus*	*bisporus*	*Psathyrellaceae*	*Agaricales*	LD	13.67	1.41	0	3	1	1
**359**	*Coprinellus*	*bisporus*	*Psathyrellaceae*	*Agaricales*	LD	10.25	0.00	0	3	1	1
**358**	*Coprinellus*	*bisporus*	*Psathyrellaceae*	*Agaricales*	LD	12.22	0.00	0	3	1	1
**305**	*Cyclocybe*	*aegerita*	*Strophariaceae*	*Agaricales*	LD	1.50	0.75	0	2	3	2
**312**	*Cyclocybe*	*aegerita*	*Strophariaceae*	*Agaricales*	LD	0.45	0.28	0	3	3	4
**496**	*Cyclocybe*	*aegerita*	*Strophariaceae*	*Agaricales*	LD	0.19	0.01	0	2	2	3
**645**	*Cyclocybe*	*erebia*	*Strophariaceae*	*Agaricales*	LD	67.50	0.94	3	4	3	3
**811**	*Cyclocybe*	*erebia*	*Strophariaceae*	*Agaricales*	LD	113.90	1.41	5	5	5	5
**530**	*Daedaleopsis*	*confragosa*	*Polyporaceae*	*Polyporales*	WR	6.28	0.08	4	5	4	4
**795**	*Daedaleopsis*	*confragosa*	*Polyporaceae*	*Polyporales*	WR	4.18	0.04	3	5	3	4
**423**	*Fayodia*	*gracilipes*	*Tricholomataceae*	*Agaricales*	LD	1.26	0.09	0	1	1	1
**805**	*Fayodia*	*gracilipes*	*Tricholomataceae*	*Agaricales*	LD	3.06	0.28	0	1	1	1
**455**	*Flammula*	*alnicola*	*Strophariaceae*	*Agaricales*	WR	17.18	4.88	3	4	2	1
**836**	*Flammulina*	*velutipes*	*Physalacriaceae*	*Agaricales*	WR	0.23	0.33	0	4	0	4
**365**	*Flammulina*	*velutipes*	*Physalacriaceae*	*Agaricales*	WR	0.93	0.56	0	5	0	5
**262**	*Fomitiporia*	*mediterranea*	*Hymenochaetaceae*	*Hymenochaetales*	WR	10.51	16.42	3	3	3	3
**565**	*Ganoderma*	*applanatum*	*Ganodermataceae*	*Polyporales*	WR	8.55	5.01	0	2	1	1
**555**	*Ganoderma*	*applanatum*	*Ganodermataceae*	*Polyporales*	WR	5.38	2.06	0	2	3	3
**556**	*Ganoderma*	*applanatum*	*Ganodermataceae*	*Polyporales*	WR	12.97	6.56	0	3	2	1
**707**	*Ganoderma*	*applanatum*	*Ganodermataceae*	*Polyporales*	WR	9.14	6.76	0	2	1	1
**705**	*Ganoderma*	*applanatum*	*Ganodermataceae*	*Polyporales*	WR	16.61	7.04	1	2	2	2
**746**	*Ganoderma*	*lucidum*	*Ganodermataceae*	*Polyporales*	WR	0.28	1.03	0	3	0	2
**522**	*Hapalopilus*	*croceus*	*Polyporaceae*	*Polyporales*	WR	4.50	0.47	0	3	0	1
**663**	*Hericium*	*coralloides*	*Hericiaceae*	*Russulales*	WR	10.59	1.03	1	1	1	1
**548**	*Hericium*	*coralloides*	*Hericiaceae*	*Russulales*	WR	44.04	0.72	2	3	2	2
**654**	*Hericium*	*erinaceus*	*Hericiaceae*	*Russulales*	WR	21.08	17.55	0	1	0	1
**374**	*Hohenbuehelia*	*auriscalpium*	*Pleurotaceae*	*Agaricales*	WR	69.41	0.19	0	2	1	2
**373**	*Hohenbuehelia*	*auriscalpium*	*Pleurotaceae*	*Agaricales*	WR	33.43	0.00	0	1	0	1
**381**	*Hypholoma*	*fasciculare*	*Strophariaceae*	*Agaricales*	WR	16.16	0.19	1	3	3	4
**362**	*Hypholoma*	*fasciculare*	*Strophariaceae*	*Agaricales*	WR	10.39	0.11	0	1	0	1
**362**	*Hypholoma*	*fasciculare*	*Strophariaceae*	*Agaricales*	WR	11.47	0.08	0	2	2	1
**703**	*Inocutis*	*dryophila*	*Hymenochaetaceae*	*Hymenochaetales*	WR	1.04	4.88	3	4	3	3
**559**	*Inonotus*	*obliquus*	*Hymenochaetaceae*	*Hymenochaetales*	WR	4.87	16.79	0	4	3	3
**694**	*Irpex*	*lacteus*	*Meruliaceae*	*Polyporales*	WR	0.15	0.20	0	1	0	1
**369**	*Irpex*	*lacteus*	*Meruliaceae*	*Polyporales*	WR	0.31	0.19	0	2	0	2
**553**	*Ischnoderma*	*benzoinum*	*Fomitopsidaceae*	*Polyporales*	WR	79.88	8.35	5	5	5	5
**656**	*Ischnoderma*	*benzoinum*	*Fomitopsidaceae*	*Polyporales*	WR	55.13	3.19	4	5	5	5
**561**	*Laetiporus*	*sulphureus*	*Fomitopsidaceae*	*Polyporales*	BR	0.14	0.00	0	4	0	4
**681**	*Laetiporus*	*sulphureus*	*Fomitopsidaceae*	*Polyporales*	BR	0.10	0.00	0	2	0	2
**389**	*Lentinula*	*edodes*	*Omphalotaceae*	*Agaricales*	WR	2.69	2.63	2	4	3	3
**390**	*Lentinula*	*edodes*	*Omphalotaceae*	*Agaricales*	WR	2.08	1.05	2	3	2	3
**391**	*Lentinula*	*edodes*	*Omphalotaceae*	*Agaricales*	WR	3.46	7.51	5	5	4	4
**724**	*Lentinula*	*edodes*	*Omphalotaceae*	*Agaricales*	WR	5.43	2.75	3	4	3	3
**590**	*Lenzites*	*tricolor*	*Polyporaceae*	*Polyporales*	WR	3.39	0.00	5	5	4	4
**797**	*Lepista*	*irina*	*Tricholomataceae*	*Agaricales*	LD	4.13	0.28	0	1	0	1
**838**	*Lepista*	*irina*	*Tricholomataceae*	*Agaricales*	LD	1.08	0.75	0	1	0	1
**394**	*Lepista*	*nuda*	*Tricholomataceae*	*Agaricales*	LD	0.48	0.09	0	2	0	1
**761**	*Lepista*	*sordida*	*Tricholomataceae*	*Agaricales*	LD	7.25	3.00	0	1	1	1
**761**	*Lepista*	*sordida*	*Tricholomataceae*	*Agaricales*	LD	9.23	5.35	0	3	1	2
**842**	*Leucoagaricus*	*bresadolae*	*Agaricaceae*	*Agaricales*	LD	56.05	1.13	0	4	3	4
**802**	*Leucoagaricus*	*bresadolae*	*Agaricaceae*	*Agaricales*	LD	93.47	1.41	0	3	2	3
**405**	*Lycoperdon*	*perlatum*	*Agaricaceae*	*Agaricales*	LD	1.67	0.28	3	2	2	1
**516**	*Lycoperdon*	*perlatum*	*Agaricaceae*	*Agaricales*	LD	2.18	1.69	5	5	5	5
**439**	*Mucidula*	*mucida*	*Physalacriaceae*	*Agaricales*	WR	12.93	5.72	4	5	4	5
**816**	*Mycena*	*crocata*	*Mycenaceae*	*Agaricales*	LD	1.89	0.00	0	2	1	1
**817**	*Mycena*	*polygramma*	*Mycenaceae*	*Agaricales*	LD	12.66	0.23	0	1	0	1
**419**	*Mycena*	*polygramma*	*Mycenaceae*	*Agaricales*	LD	15.54	0.00	0	1	0	1
**520**	*Mycena*	*polygramma*	*Mycenaceae*	*Agaricales*	LD	10.93	0.84	0	1	0	1
**623**	*Mycetinis*	*alliaceus*	*Omphalotaceae*	*Agaricales*	LD	0.65	16.7	0	2	0	2
**343**	*Omphalina*	*mutila*	*Tricholomataceae*	*Agaricales*	LD	17.69	20.36	0	4	4	4
**388**	*Omphalotus*	*japonicus*	*Omphalotaceae*	*Agaricales*	WR	0.39	0.84	0	3	0	2
**708**	*Onnia*	*tomentosa*	*Hymenochaetaceae*	*Hymenochaetales*	WR	6.09	14.92	0	3	0	2
**616**	*Oxyporus*	*latemarginatus*	*Schizoporaceae*	*Hymenochaetales*	WR	3.70	12.57	3	5	5	5
**810**	*Oxyporus*	*latemarginatus*	*Schizoporaceae*	*Hymenochaetales*	WR	1.55	7.69	5	5	5	5
**615**	*Oxyporus*	*latemarginatus*	*Schizoporaceae*	*Hymenochaetales*	WR	1.16	10.23	3	4	4	4
**276**	*Phellinus*	*hartigii*	*Hymenochaetaceae*	*Hymenochaetales*	WR	0.32	0.00	0	5	0	3
**575**	*Phellinus*	*igniarius*	*Hymenochaetaceae*	*Hymenochaetales*	WR	0.45	0.94	0	3	0	3
**577**	*Phellinus*	*igniarius*	*Hymenochaetaceae*	*Hymenochaetales*	WR	0.23	2.20	0	1	0	1
**269**	*Phellinus*	*igniarius*	*Hymenochaetaceae*	*Hymenochaetales*	WR	0.51	6.29	0	5	5	5
**657**	*Phellinus*	*igniarius*	*Hymenochaetaceae*	*Hymenochaetales*	WR	1.45	2.35	0	3	2	3
**758**	*Phellinus*	*igniarius*	*Hymenochaetaceae*	*Hymenochaetales*	WR	2.93	0.84	0	1	1	1
**274**	*Phellinus*	*igniarius*	*Hymenochaetaceae*	*Hymenochaetales*	WR	4.50	1.97	0	1	1	1
**265**	*Phelinus*	*pomaceus*	*Hymenochaetaceae*	*Hymenochaetales*	WR	1.16	13.32	3	3	1	1
**587**	*Phellinus*	*robustus*	*Hymenochaetaceae*	*Hymenochaetales*	WR	8.71	16.14	0	3	3	2
**715**	*Phlebia*	*chrysocreas*	*Meruliaceae*	*Polyporales*	WR	7.93	0.00	0	1	0	1
**846**	*Pholiota*	*adiposa*	*Strophariaceae*	*Agaricales*	WR	3.57	10.04	3	4	2	3
**847**	*Pholiota*	*adiposa*	*Strophariaceae*	*Agaricales*	WR	1.09	2.68	2	1	2	1
**683**	*Pholiota*	*adiposa*	*Strophariaceae*	*Agaricales*	WR	1.95	4.41	3	3	2	3
**780**	*Pholiota*	*aurivella*	*Strophariaceae*	*Agaricales*	WR	56.08	10.70	4	4	3	3
**849**	*Pholiota*	*aurivella*	*Strophariaceae*	*Agaricales*	WR	45.58	9.05	3	3	3	2
**450**	*Pleurotus*	*calyptratus*	*Pleurotaceae*	*Agaricales*	WR	1.21	0.28	0	3	2	1
**691**	*Pleurotus*	*citrinopileatus*	*Pleurotaceae*	*Agaricales*	WR	5.50	6.85	0	4	0	1
**564**	*Pleurotus*	*cornucopiae*	*Pleurotaceae*	*Agaricales*	WR	2.16	0.38	0	3	1	4
**464**	*Pleurotus*	*cornucopiae*	*Pleurotaceae*	*Agaricales*	WR	3.08	1.13	2	2	3	3
**466**	*Pleurotus*	*cystidiosus*	*Pleurotaceae*	*Agaricales*	WR	13.3	9.19	0	2	2	3
**461**	*Pleurotus*	*djamor*	*Pleurotaceae*	*Agaricales*	WR	31.08	1.78	4	4	3	3
**666**	*Pleurotus*	*djamor*	*Pleurotaceae*	*Agaricales*	WR	88.86	4.50	5	5	5	5
**468**	*Pleurotus*	*dryinus*	*Pleurotaceae*	*Agaricales*	WR	14.04	13.89	0	1	1	1
**372**	*Pleurotus*	*eryngii*	*Pleurotaceae*	*Agaricales*	WR	1.24	2.35	0	3	0	1
**754**	*Pleurotus*	*eryngii*	*Pleurotaceae*	*Agaricales*	WR	1.08	1.03	0	1	0	1
**408**	*Pleurotus*	*eryngii*	*Pleurotaceae*	*Agaricales*	WR	2.40	3.38	0	3	1	3
**354**	*Pleurotus*	*eryngii*	*Pleurotaceae*	*Agaricales*	WR	0.29	0.19	0	1	0	1
**544**	*Pleurotus*	*eryngii*	*Pleurotaceae*	*Agaricales*	WR	1.38	4.22	0	1	2	2
**819**	*Pleurotus*	*eryngii*	*Pleurotaceae*	*Agaricales*	WR	1.45	4.32	0	3	2	3
**625**	*Pleurotus*	*eryngii*	*Pleurotaceae*	*Agaricales*	WR	1.01	0.28	0	2	0	2
**830**	*Pleurotus*	*eryngii*	*Pleurotaceae*	*Agaricales*	WR	0.23	2.25	0	1	0	1
**843**	*Pleurotus*	*eryngii*	*Pleurotaceae*	*Agaricales*	WR	4.42	3.50	0	3	3	3
**692**	*Pleurotus*	*ostreatus*	*Pleurotaceae*	*Agaricales*	WR	66.78	1.13	4	4	4	4
**462**	*Pleurotus*	*ostreatus*	*Pleurotaceae*	*Agaricales*	WR	106.68	6.94	5	5	3	3
**473**	*Pleurotus*	*ostreatus*	*Pleurotaceae*	*Agaricales*	WR	72.95	6.94	3	4	5	5
**766**	*Pleurotus*	*ostreatus*	*Pleurotaceae*	*Agaricales*	WR	50.37	2.28	2	3	3	3
**479**	*Pleurotus*	*pulmonarius*	*Pleurotaceae*	*Agaricales*	WR	47.49	1.03	5	5	3	3
**589**	*Polyporus*	*brumalis*	*Polyporaceae*	*Polyporales*	WR	0.03	15.48	0	2	1	1
**818**	*Polyporus*	*brumalis*	*Polyporaceae*	*Polyporales*	WR	0.01	10.00	0	2	0	2
**591**	*Polyporus*	*ciliatus*	*Polyporaceae*	*Polyporales*	WR	8.27	2.72	0	1	2	1
**598**	*Polyporus*	*lepideus*	*Polyporaceae*	*Polyporales*	WR	2.29	1.16	3	2	3	2
**608**	*Polyporus*	*lepideus*	*Polyporaceae*	*Polyporales*	WR	4.69	0.14	5	5	5	5
**534**	*Polyporus*	*lepideus*	*Polyporaceae*	*Polyporales*	WR	3.04	0.28	3	2	3	2
**676**	*Polyporus*	*squamosus*	*Polyporaceae*	*Polyporales*	WR	0.37	1.29	0	2	0	2
**261**	*Porodaedalea*	*pini*	*Hymenochaetaceae*	*Hymenochaetales*	WR	1.27	0.56	3	3	3	3
**735**	*Porodaedalea*	*pini*	*Hymenochaetaceae*	*Hymenochaetales*	WR	0.91	1.26	3	3	2	2
**261**	*Porodaedalea*	*pini*	*Hymenochaetaceae*	*Hymenochaetales*	WR	1.25	0.38	2	2	2	2
**492**	*Psilocybe*	*subaeruginosa*	*Strophariaceae*	*Agaricales*	LD	10.20	0.19	0	1	1	1
**488**	*Psilocybe*	*subaeruginosa*	*Strophariaceae*	*Agaricales*	LD	14.44	0.28	0	1	1	1
**595**	*Pycnoporus*	*sanguineus*	*Polyporaceae*	*Polyporales*	WR	33.77	3.03	4	5	5	5
**596**	*Pycnoporus*	*sanguineus*	*Polyporaceae*	*Polyporales*	WR	56.92	9.01	5	5	5	5
**702**	*Rhodocollybia*	*butyracea*	*Omphalotaceae*	*Agaricales*	LD	54.6	0.28	0	1	2	1
**349**	*Rhodocollybia*	*maculata*	*Omphalotaceae*	*Agaricales*	LD	4.39	0.19	0	1	0	1
**110**	*Serpula*	*himantioides*	*Serpulaceae*	*Boletales*	BR	0.00	0.00	0	5	0	5
**752**	*Schizophyllum*	*commune*	*Schizophyllaceae*	*Agaricales*	WR	0.14	0.47	0	5	0	4
**658**	*Sparassis*	*crispa*	*Sparassidaceae*	*Polyporales*	BR	0.00	0.00	0	1	0	1
**524**	*Stereum*	*gausapatum*	*Stereaceae*	*Russulales*	WR	0.39	0.19	0	3	0	3
**610**	*Trametes*	*hirsuta*	*Polyporaceae*	*Polyporales*	WR	24.23	5.54	5	5	5	5
**611**	*Trametes*	*versicolor*	*Polyporaceae*	*Polyporales*	WR	2.85	7.88	5	5	3	4
**614**	*Trametes*	*versicolor*	*Polyporaceae*	*Polyporales*	WR	25.05	6.38	5	5	5	5
**528**	*Trametes*	*versicolor*	*Polyporaceae*	*Polyporales*	WR	47.84	11.82	4	5	4	5
**704**	*Trametes*	*versicolor*	*Polyporaceae*	*Polyporales*	WR	3.56	7.60	4	5	4	5
**612**	*Trametes*	*versicolor*	*Polyporaceae*	*Polyporales*	WR	63.54	1.60	5	5	5	5
**750**	*Tricholoma*	*sejunctum*	*Tricholomataceae*	*Agaricales*	LD	11.24	0.00	5	5	5	5
**673**	*Tyromyces*	*chioneus*	*Polyporaceae*	*Polyporales*	WR	0.54	4.32	0	2	2	2
**267**	*Tyromyces*	*chioneus*	*Polyporaceae*	*Polyporales*	WR	0.85	3.57	2	3	2	3
**277**	*Tyromyces*	*chinoeus*	*Polyporaceae*	*Polyporales*	WR	0.12	1.13	0	1	0	1

^1^ Laccase activity in maximum of production; 10^−3^ U.ml^−1^; ^2^ MnP activity in maximum of production; 10^−4^ U.ml^−1^; ^3^ Decolorized zone after 14 days of cultivation: 0−20 mm = 1; 21−30 mm = 2; 31−50 mm = 3; 51−70 mm = 4; and 71−90 mm = 5; ^4^ Diameter of mycelia after 14 days of cultivation: 0−20% of control = 1; 21−30% of control = 2; 31−50% of control = 3; 51−70% of control = 4; 71−100% of control = 5; and control = diameter of mycelia after 14 days of cultivation in each strain on medium without dyes.

**Table 5:** Distribution of the strains with high decolorization capacity and with high ligninolytic enzyme production with respect to their taxonomic classification.

Number of Strains (*n*)								
Order	*n*	Family	*n*	High Ligninolytic Enzyme Activity and Decolorization Capacity				
				Laccase	MnP	Orange G	RBBR	Orange G and RBBR
*Agaricales*	85	*Agaricaceae*	5	2 ^a^	0 ^b^	2/1 ^c^	2/1 ^c^	1/1 ^c^
*Agaricales*		*Entolomataceae*	3	3	3	3/0	1/0	1/0
*Agaricales*		*Mycenaceae*	4	0	0	0/0	0/0	0/0
*Agaricales*		*Omphalotaceae*	8	1	2	2/1	3/0	2/0
*Agaricales*		*Phanerochaeteceae*	1	0	0	0/0	0/0	0/0
*Agaricales*		*Physalacriaceae*	7	0	1	1/0	1/0	1/0
*Agaricales*		*Pleurotaceae*	24	9	10	6/3	9/2	6/1
*Agaricales*		*Psathyrellaceae*	4	0	0	0/0	0/0	0/0
*Agaricales*		*Schizophyllaceae*	1	0	0	0/0	0/0	0/0
*Agaricales*		*Strophariaceae*	19	5	5	8/1	9/1	5/1
*Agaricales*		*Tricholomataceae*	9	0	2	1/1	2/1	1/1
*Hymenochaetales*	19	*Hymenochaetaceae*	16	0	7	5/0	3/0	3/0
*Hymenochaetales*		*Schizoporaceae*	3	0	3	3/1	3/2	3/1
*Polyporales*	41	*Fomitopsidaceae*	7	2	2	2/1	2/2	2/1
*Polyporales*		*Ganodermataceae*	6	0	4	0/0	0/0	0/0
*Polyporales*		*Meruliaceae*	5	2	0	2/1	2/1	2/1
*Polyporales*		*Polyporaceae*	22	4	11	14/7	14/6	14/5
*Polyporales*		*Sparassidaceae*	1	0	0	0/0	0/0	0/0
*Russulales*	4	*Hericiaceae*	3	1	1	0/0	0/0	0/0
*Russulales*		*Stereaceae*	1	0	0	0/0	0/0	0/0

^a^ The number of strains exhibiting high laccase activity (peak activity > 3.10^−2^ U.ml^−1^); ^b^ The number of strains exhibiting high MnP activity (peak activity > 3.10^−4^ U.ml^−1^); ^c^ The number of strains exhibiting high/highest decolorization capacity after 14 days of cultivation (decolorized zone 31–90 mm/71–90 mm).
